# Continuation of CDK4/6 Inhibition and Switching of Hormonal Therapy After Progression on Prior CDK4/6 Inhibitors in HR+/HER2- Breast Cancer: A Systematic Review and Meta-Analysis

**DOI:** 10.7759/cureus.73738

**Published:** 2024-11-15

**Authors:** Allan Ramos-Esquivel, Isaac Ramírez-Jiménez, Alvaro Víquez-Jaikel

**Affiliations:** 1 School of Medicine, Department of Pharmacology, Universidad de Costa Rica, San José, CRI; 2 School of Medicine, Universidad de Costa Rica, San Pedro, CRI; 3 Department of Pharmacy, Hospital San Juan de Dios, Caja Costarricense de Seguro Social, San José, CRI

**Keywords:** abemaciclib, breast cancer, cyclin-dependent kinase 6, palbociclib, ribociclib

## Abstract

This study aims to determine the efficacy of maintaining cyclin-dependent kinase 4/6 (CDK4/6) inhibition and switching endocrine therapy (ET) versus ET alone after progression on prior CDK4/6 inhibitors (CDK4/6i) in patients with hormone-receptor-positive, human epidermal growth factor receptor-2-negative breast cancer. We identified phase II and III comparative randomized clinical trials through a systematic search across relevant clinical databases. A random effects model was used to determine the pooled hazard ratio (HR) for progression-free survival (PFS) according to the inverse-variance method. Heterogeneity was measured using tau^2^ and I^2^ statistics. The pooled odds ratio for the overall response rate was calculated through the Mantel-Haenszel method in a random effects model. A narrative review was done to describe treatment-related side effects. After the systematic search, we identified four trials (n=833) that accomplished the inclusion criteria. Switching ET and maintaining CDK4/6 inhibition was associated with longer PFS than switching the hormonal therapy alone (HR: 0.77; 95% CI: 0.60-0.99; p=0.04) with moderate heterogeneity among the included trials (tau^2^: 0.04; I^2^: 56%; p=0.08). Subgroup analysis identified a PFS benefit from this approach independently of the length of previous CDK4/6 inhibition. The PFS benefit was more pronounced in those individuals who received abemaciclib or ribociclib as second CDK4/6i. Continuation of CDK4/6 inhibition was associated with higher rates of grade 3 and 4 neutropenia (range: 25-40%) and anemia (range: 1.7-11%). In conclusion, switching CDK4/6i and ET conferred a statistically significant improvement of PFS in comparison to ET alone in patients with progression or recurrence on prior CDK4/6i-containing therapy.

## Introduction and background

Breast cancer (BC) is one of the most frequently diagnosed cancers worldwide and the leading cause of death due to cancer among female patients [1]. Approximately 70-80% of invasive BC belongs to the hormone receptor-positive (HR+) and human epidermal growth factor receptor-2-negative (HER2-) subtype [2]. For these patients, endocrine therapy (ET) has been the cornerstone of management in the adjuvant and metastatic setting. Specifically, tamoxifen, aromatase inhibitors (i.e., letrozole, anastrozole, exemestane), and fulvestrant (a selective estrogen receptor downregulator) have been used for decades to treat this specific type of BC. [2] However, despite surgery, radiotherapy, chemotherapy, and ET, a subset of patients experience systemic relapse, while some patients are diagnosed with *de novo* metastatic disease. For this particular population, the median overall survival (OS) is currently five years [1]. Hence, new therapies have been explored to increase disease control and prolong survival, especially in the metastatic setting due to its high morbidity and mortality.

The proven efficacy of cyclin-dependent kinase 4/6 (CDK4/6) inhibitors (CDK4/6i) in combination with ET in the adjuvant [3] and metastatic setting [4-6] has led to the approval of some of these agents in such clinical scenarios. CDK4/6 inhibitors block the proliferation of BC cells by inducing a G1 cell cycle arrest and promoting apoptosis [7]. It has been demonstrated that CDK4/6 can also alter cellular metabolism, depleting antioxidants and increasing reactive oxygen species, while inducing cell senescence [7].

The use of CDK4/6i (abemaciclib, palbociclib, and ribociclib) plus ET as first-line therapy is considered the standard of therapy in patients with metastatic BC due to clinically meaningful benefits in terms of progression-free survival (PFS) [4,6], OS [8], time-to-chemotherapy [9], and quality of life [9]. However, most patients will eventually progress on CDK4/6i and ET due to acquired or intrinsic resistance by several mechanisms involving the retinoblastoma (Rb) protein pathway (loss of Rb function), or p16, CDK6, AURKA, and CDK4 amplifications. Other bypass mechanisms include the phosphatidylinositol 3-kinase (PI3K), Akt, and mTOR (PAM) pathway, overexpression of the fibroblast growth factor receptor (FGFR), mutations in the estrogen signaling, such as the estrogen receptor 1 (ESR1) mutations, or other gene aberrations affecting cell cycle progression [10]. In this particular population, the optimal sequence of treatment is an unmet clinical need.

Although some new therapies targeting the PAM pathway (such as alpelisib [11] and capivasertib [12]) or estrogen signaling [13] (i.e., elacestrant), and new antibody-drug conjugates (e.g., trastuzumab deruxtecan [14] and sacituzumab govitecan [15]) have shown a role in this scenario, recent clinical trials have also tested the hypothesis of maintaining the CDK4/6 inhibition and switching the ET as a way to overcome the aforementioned resistance mechanisms that led to clinical progression [16-19]. Given the poor prognosis of patients after first-line treatment failure despite the broad availability of systemic therapy [2,7], it is mandatory to explore second-line therapies that could significantly improve OS while preserving quality of life. Therefore, this systematic review and meta-analysis aim to summarize the clinical evidence and assess the efficacy of maintaining the CDK4/6 inhibition and switching the ET as second-line therapy for patients progressing on previous CDK4/6i.

## Review

Methods

Search Strategy and Study Selection

Two authors (AVJ and IRJ) independently examined the abstracts and titles of the clinical trials retrieved from the systematic search done in electronic databases (EMBASE, MEDLINE, OVID, the Cochrane Central Register of Control Trials, and Web of Science) from January 2017 to July 2024 (see the Appendix file for the detailed search strategy). The search was carried out in July 2024 following a designed protocol using the PICO (population, intervention, control, and outcomes) framework. In cases of duplications of the same clinical trial, only the most recent follow-up was included. The authors examined full-text articles of potentially eligible trials for compliance with the eligibility criteria. The corresponding author of each trial was contacted if the reported clinical trials did not include all the necessary data for analyses. Disagreements were resolved in consultation with a third author (ARE) and resolved by consensus. Data from selected studies were tabulated in tables, which included information on trial design, participants, interventions, and outcomes.

Eligibility Criteria

We only included data from published randomized clinical trials (either in a complete or abstract form) with HR+/HER2- BC patients who progressed on treatment with abemaciclib, palbociclib, or ribociclib plus ET in the metastatic setting or within 12 months of CDK4/6i exposure in the adjuvant setting. We excluded observational studies, non-comparative studies, and trials published in non-English languages. Additionally, studies with no abstract available and trials exploring combination therapy other than ET and CDK4/6i were excluded. We summarized the safety of each arm in all patients who received at least one dose of the study treatment. These adverse drug reactions were graded according to the National Cancer Institute Common Terminology Criteria for Adverse Events (version 4.0).

Outcomes

The primary outcome was PFS, calculated from the date of randomization to the date of progression according to RECIST 1.1 criteria or death. Secondary outcomes were objective response rate (ORR), defined as the percentage of patients with complete or partial response as per RECIST 1.1, and safety.

To explore potential sources of heterogeneity and to assess the consistency of the treatment effect across different patient subgroups, we performed a pre-specified subgroup analysis based on the type of CDK4/6i used after failure to first-line or adjuvant therapy, the duration of previous CDK4/6 inhibition (more or less than 12 months), and according to the presence of ESR1 and PI3K (or Akt or PTEN) mutations, since previous studies have shown that CDK4/6 efficacy can vary depending on previous exposure to ET and according to the presence of some genetic variants [7].

Quality Assessment

Three reviewers independently assessed the risk of bias by using the Cochrane Collaboration Tool guideline [20], including adequate sequence generation, adequate allocation concealment, blinding, incomplete outcome data addressed, and freedom from selective reporting. Publication bias was visually examined in a funnel plot for the primary outcome. The risk of bias was considered as ‘low risk’, ‘high risk’, or ‘unclear risk’, as suggested by the Cochrane Collaboration Tool guideline [20]. Discrepancies were resolved by consensus.

Data Collection and Statistical Analysis

The pooled hazard ratio and its 95% confidence interval (95%CI) were used to describe treatment efficacy. A random effects model (DerSimonian-Laird method), according to the inverse-variance method (as described by Parmar et al. [21]), was chosen because some degree of heterogeneity in the effect sizes was assumed.

For the association of ORR among group comparisons, we employed the Mantel-Haenszel odds ratio (OR) and its corresponding 95%CI through a random effects model. Heterogeneity was determined by the tau^2^ and I^2^ statistics. No sensitivity analyses were conducted to explore heterogeneity due to the lack of data. Data analyses were performed using RevMan (version 5.3; Cochrane Collaboration, London, UK) software. The Preferred Reporting Items for Systematic Review and Meta-Analysis (PRISMA) statement for reporting systematic reviews was followed [22].

Role of Funding Source

No funding source had any role in the study design, data collection, data analysis, data interpretation, or writing of this manuscript.

Results

Study Selection

**Figure 1:**
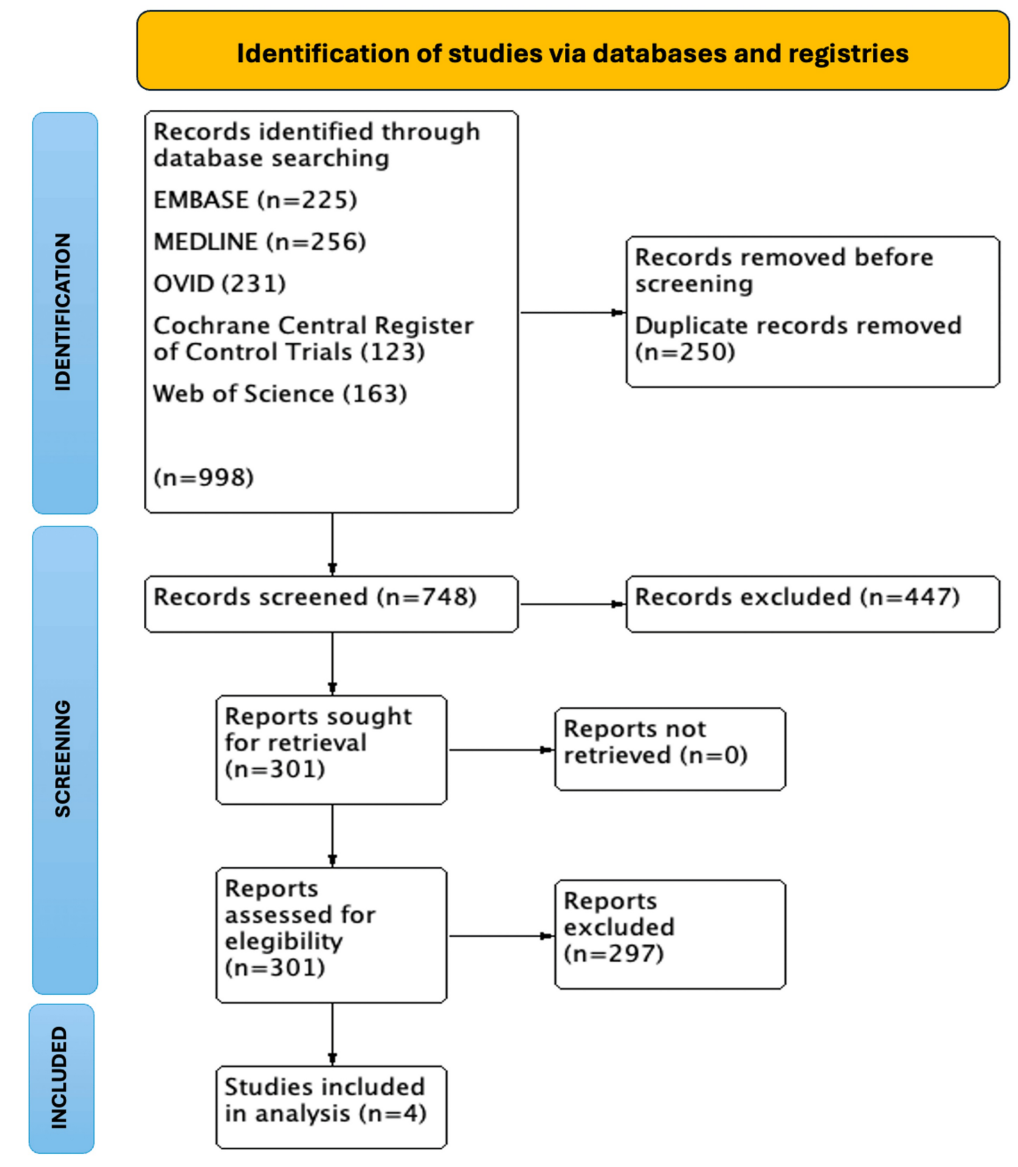
Preferred Reporting Items for Systematic Reviews and Meta-Analyses (PRISMA) flow diagram

After applying the search strategy, we identified four studies (one phase III and three phase II randomized clinical trials) that explored the continuation of CDK inhibition in addition to ET versus ET alone after failure on CDK4/6i and hormonal therapy (n=833). Figure 1 summarizes the review process according to the PRISMA flow diagram for literature search.

**Figure 2:**
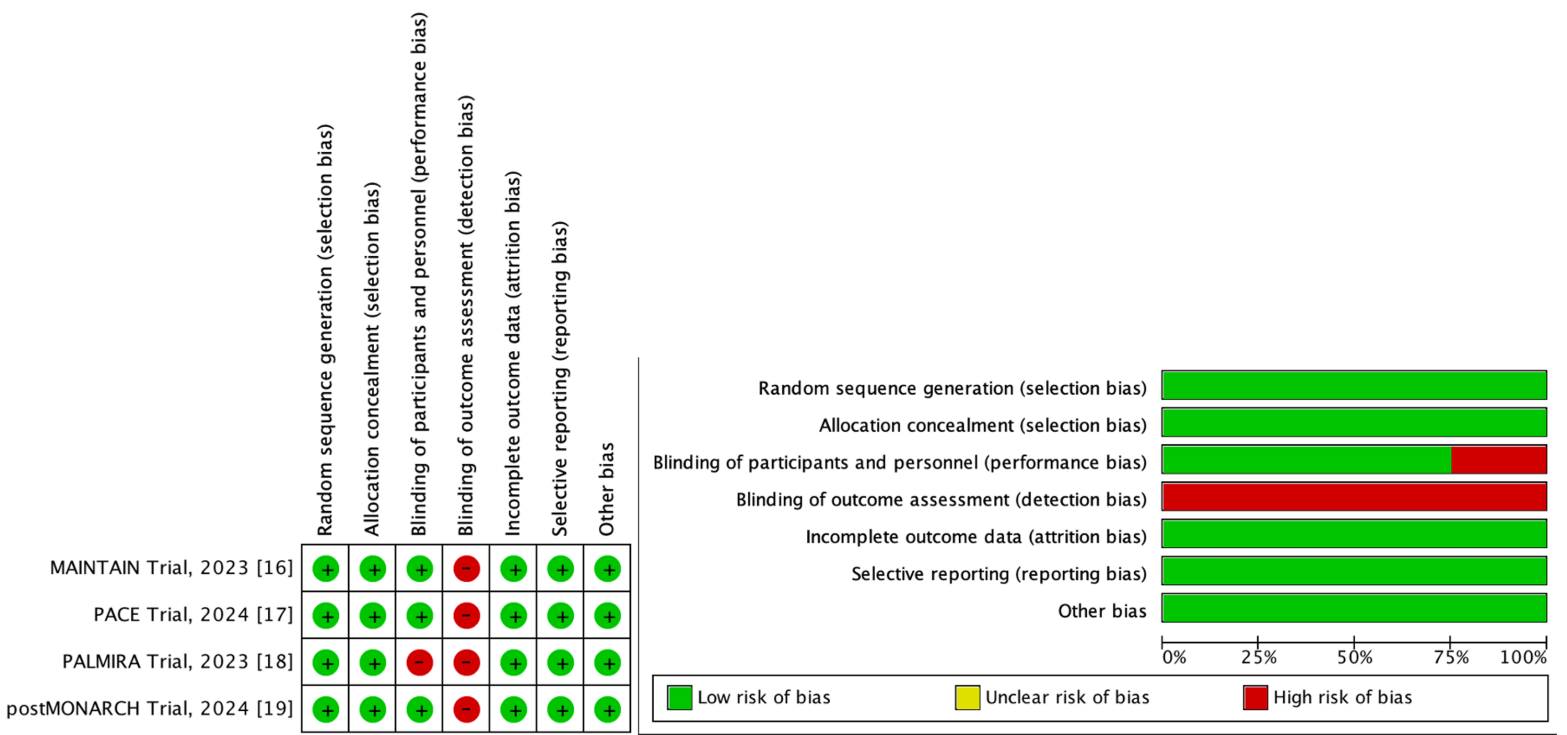
Risk of bias assessment of the included studies Source: Refs. [16-19]

An assessment of the risk of bias found in these trials is shown in Figure 2. Of note, all included trials had a high risk of detection bias because of the lack of blinding of assessors, while the PALMIRA trial [18] was an open-label study with a considered high risk of performance bias.

Description of Studies and Patients

In two of the selected trials, patients continued with the same CDK4/6 inhibitor (palbociclib), while the remaining studies included a majority of patients that changed the type of CDK4/6i (with either ribociclib or abemaciclib). Table 1 provides a comparison among the included clinical trials in terms of enrolled patients, interventions, and outcomes (Table 2). Of note, palbociclib was the most common CDK4/6i employed in the first-line setting (range: 59%-100%), and fulvestrant was the most used ET employed as a comparator. In addition, most patients received the first CDK4/6i for more than 12 months as anti-metastatic therapy, and a minority of patients (from 14% to 33%) progressed during the first year of CDK4/6i therapy.

**Table 1:** General characteristics of the included trials CDKi: cyclin-dependent kinase 4/6 inhibitor *The PACE study included a total of 220 patients, including a third arm (n ¼ 54) with avelumab plus palbociclib plus fulvestrant. Source: Refs. [16-19]

Trial	Phase	Sample size (randomization)	Statistical power	Median follow-up (mo)	CDK4/6i	Comparison arms	Initial CDK4/6i	Response to prior CDK4/6i	Previous use of chemotherapy in the metastatic setting	Patients with CDK4/6i beyond 12 mo as first-line or adjuvant therapy	Primary endpoint
Kalinsky et al. (MAINTAIN) [16]	II	119 (1:1)	80%	18.2	Ribociclib 600 mg once daily 3 weeks on, 1 week off	Ribociclib +Fulvestrant/Exemestane vs Fulvestrant (83%) or Exemestane (17%)	Palbociclib (86.5%) Ribociclib (11.7%) Abemaciclib (1.7%)	Any (only patients with metastatic or unresectable disease allowed)	9.2%	67%	Progression-free survival
Mayer et al. (PACE) [17]	II	166 (2:1)	80%	23.6	Palbociclib 125 mg, once daily, 3 weeks on, 1 week off	Palbociclib+Fulvestrant vs Fulvestrant (100%)	Palbociclib (90.9%) Ribociclib (4.5%) Abemaciclib (4.1%)	At least 6 months in the metastatic setting or within 12 mo in the adjuvant setting	16.3%	75.9%	Progression-free survival
Llombart-Cussac et al. (PALMIRA) [18]	II	198 (2:1)	80%	13.2	Palbociclib 75/100/125 mg, once daily, 3 weeks on, 1 week off	Palbociclib+Fulvestrant/Letrozole vs Fulvestrant (90%) or Letrozol (10%)	Palbociclib (100%)	At least 24 weeks with clinical benefit in the metastatic setting or at least 12 mo.of adjuvant treatment but no more than12 mo. from completion.	Not allowed	85.3%	Progression-free survival
Kalinsky et al. (PostMONARCH) [19]	III	368 (1:1)	80%	NR	Abemaciclib 150 mg daily	Abemaciclib+Fulvestrant vs Fulvestrant (100%)	Palbociclib (59%) Ribociclib (33%) Abemaciclib (8%)	Any	Not allowed	74%	Progression-free survival

**Table 2:** Overall results of the included trials CI: Confidence Interval; ECOG: Eastern Cooperative Oncology Group performance status; ET: Endocrine Therapy; HR: Hazard Ratio; IQR: Interquartile Range; mPFS: Median Progression-Free Survival; mOS: Median Overall Survival; NR: Not Reported

Trial	MAINTAIN [16]		PACE [17]		PALMIRA [18]		PostMONARCH [19]	
Comparison arms	Ribociclib + ET (n=60)	ET (n=59)	Palbociclib + ET (n=111)	ET (n=55)	Palbociclib + ET (n=136)	ET (n=62)	Abemaciclib + ET (n=182)	ET (n=186)
Median age in years (range)	55 (IQR:48-67)	59 (IQR:51-65)	55 (28-77)	58 (36-77)	59 (33-85)	61 (34-83)	58 (27-86)	61 (28-85)
Postmenopausal	100	100	78.4	85.5	86.8	90.3	NR	NR
ECOG 0 (%)	66.7	64.4	NR	NR	66.2	50	57	58
ECOG 1 (%)	33.3	35.6	NR	NR	33.1	50	43	43
Visceral involvement (%)	60	59.3	63.1	52.7	61.8	59.7	62	59
Measurable disease at baseline (%)	58.3	59.3	65.8	67.3	69.1	71.0	72	68
Progression-free survival	HR: 0.57 (95%CI: 0.39-0.85); p=0.006; mPFS: 5.3 vs 2.8 mo		HR: 1.11 (90%CI: 0.79-1.55); p=0.62; mPFS: 4.6 vs 4.8 mo		HR: 0.84 (95%CI: 0.66-1.07); p=0.149; mPFS: 4.9 vs 3.6 mo		HR: 0.66 (95% CI: 0.48-0.91); p=0.01; mPFS: 5.6 vs 3.9 mo	
Overall response rate (95% CI)	20% (9.8-30)	11% (3.0-19.0)	9.0% (4.5-13.5)	7.3% (1.5-13.0)	4.4% (1.6-9.4)	1.6% (0-8.7)	17% (11.5-22.5)	7% (3.3-10.7)
Clinical Benefit rate (95% CI)	43% (30.5-55.5)	25% (36.0-13.9)	32.4% (25.1-39.7)	29.1% (19.0-39.2)	41.9% (33.5-50.7)	27.4% (16.9-40.2)	NR	NR
Overall survival	NR		HR: 1.02 (90%CI: 0.67-1.56); mOS: 24.6 vs 27.5 mo		HR: 1.06 (95% CI: 0.75-1.51); mOS: 28.3 vs 28.8 mo		NR	
Treatment-related adverse events grade 3 or 4 (%)								
Neutropenia	40.0	1.7	32.7	0	38.5	0	25.0	0
Anemia	1.7	1.7	4.5	0	3.0	0	11.0	4.0
Thrombocytopenia	0	0	0.9	0	NR	NR	4.0	2.0
Diarrhea	0	0	0	0	0	0	4.0	2.0
Fatigue	2.0	0	1.8	0	0	0	3.0	1.0
Nausea	0	0	0	0	0	0	3.0	0

Outcomes

**Figure 3:**
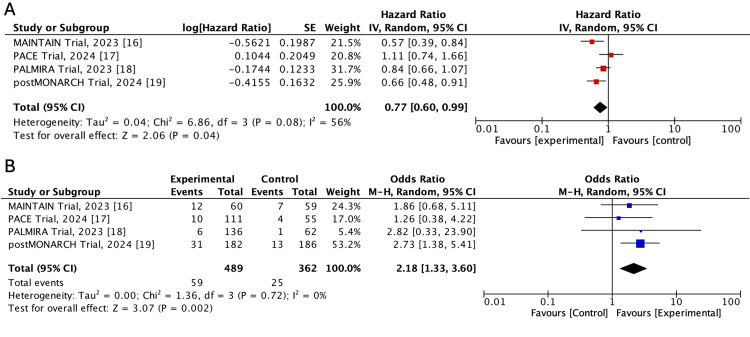
Forest plot of hazard ratios for progression-free survival (A) and odds ratios for the overall response rate from the selected trials comparing the continuation of CDK4/6 inhibition plus endocrine therapy (experimental) versus endocrine therapy alone (control) Source: Refs. [16-19]

PFS: It was the primary outcome for all the included trials (defined from the start of treatment until time to progression according to the RECIST 1.1. criteria). As shown Figure 3A, a continuation of CDK4/6i resulted in a significant increase in PFS (pooled HR: 0.77; 95%CI: 0.60-0.99; p=0.04) with moderate heterogeneity among trials (tau^2^: 0.04; I^2^: 56%; p=0.08).

Overall response rate: The odds of objective response was significantly higher with continuation of CDK4/6 inhibition (Mantel-Haenszel OR: 2.18; 95%CI: 1.33-3.60; p=0.002) in comparison to ET with fulvestrant or an aromatase inhibitor, with no significant heterogeneity found among trials (tau^2^: 0; I^2^: 0%; p=0.72) (Figure 3B).

Treatment-related adverse events: Table 2 summarizes the cumulative incidences of the most frequent serious adverse events (grade 3 or higher). Neutropenia rates were higher in the abemaciclib, palbociclib, and ribociclib arms of each included trial. The second most frequent side effect reported in these trials was anemia in the continuation of the CDK4/6i arms, ranging from 1.7 to 11%.

Subgroup Analysis

**Figure 4:**
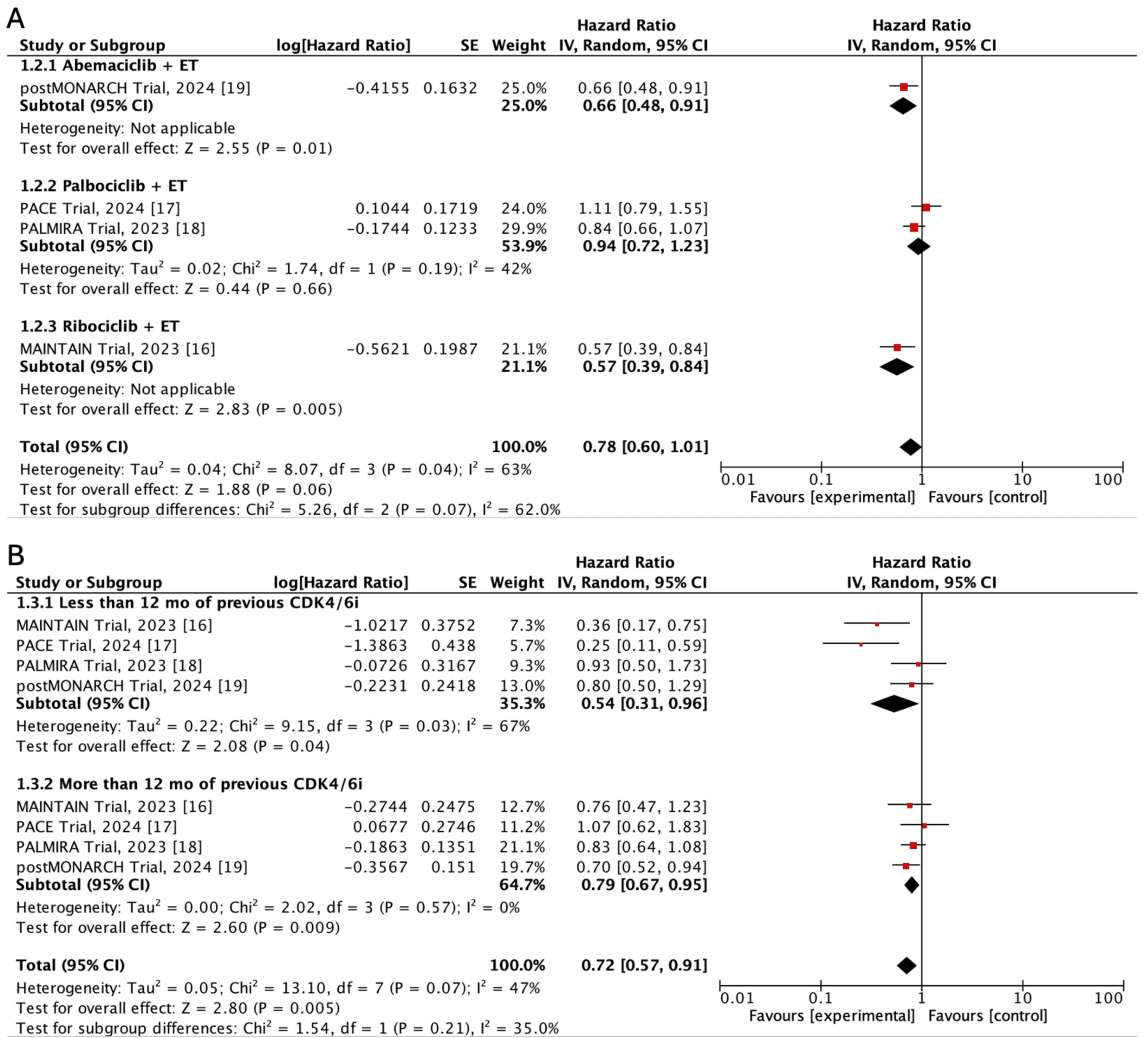
Forest plot of hazard ratios for progression-free survival according to the type (A) and length (B) of the CDK4/6 inhibitor used after failure on the previous CDK4/6 inhibition Source: Refs. [16-19]

The PFS analysis according to the type and length of previous CDK4/6 inhibition is presented in Figure 4. We found statistically significant differences in terms of PFS only in the subgroup of studies that employed abemaciclib or ribociclib as second-line therapy. In contrast, the use of palbociclib as CDK4/6i was not related to better PFS, neither in the PALMIRA nor the PACE trial (Figure 4A). Despite this finding, between-subgroup differences were not statistically significant (interaction p value=0.07). Similarly, the improvement of PFS was noticed regardless of the length of previous CDK4/6 inhibition (less or more than 12 months)(interaction p value=0.21) (Figure 4B).

**Figure 5:**
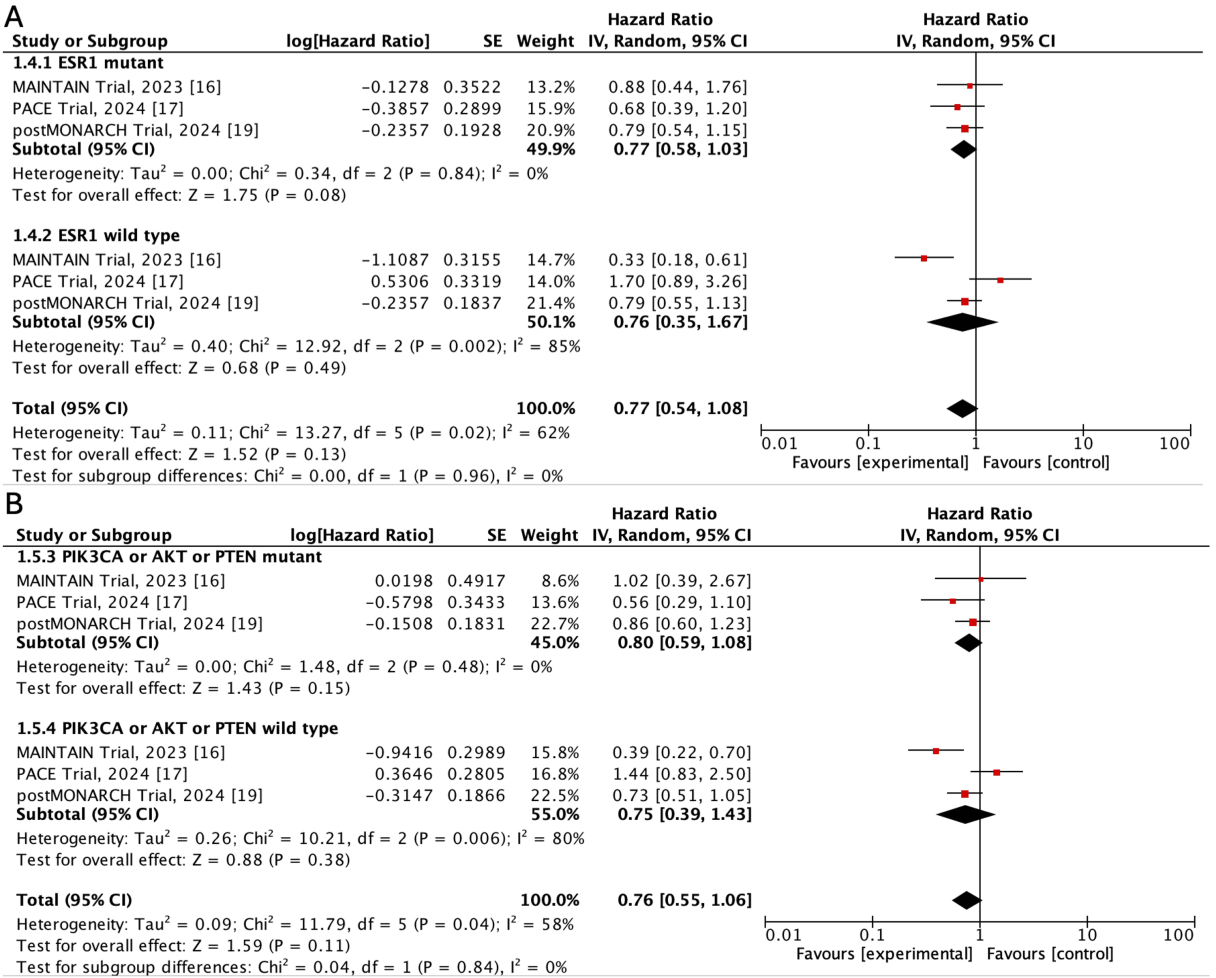
Forest plot of hazard ratios for progression-free survival according to ESR1 mutation (A) and to PI3KCA, Akt, or PTEN mutation status (B) Source: Refs. [16-19]

When patients were stratified according to mutations in the PAM pathway (PI3KCA, Akt, or PTEN) or in ESR1 (data available for only three trials), the pooled HR for PFS was not statistically significant (HR: 0.80; 95%CI: 0.59-1.08; p=0.15, and HR: 0.77; 95%CI: 0.58-1.03; p=0.08, respectively), albeit high heterogeneity between studies (I^2^: 85% and 80%, respectively) (Figure 5).

Discussion

The optimal sequence of therapy for metastatic BC patients after failure on CDK4/6i and ET has not been established [2,23]. In this systematic review and meta-analysis, we resumed the available data from phase II and III comparative clinical trials showing a PFS benefit with the continuation of CDK4/6 inhibition and switching of ET in this particular population. In summary, there was a 23% reduction in the hazard of progression or death and a two-fold increase in the odds of overall response with this approach.

The sample population of the included clinical trials was broadly homogeneous in terms of age, status performance, and tumor burden. Similarly, there were similarities regarding the use of previous therapies since most of them were rarely treated with chemotherapy, and most received an aromatase inhibitor plus palbociclib as first-line therapy in the metastatic setting, with a tumor progression after 12 months from the start of treatment. However, one key difference among the included trials was the type of CDK4/6i in the experimental arm. Although previous studies have shown some similarities in terms of efficacy among abemaciclib, palbociclib, and ribociclib in patients with advanced BC [7-9,24], clinical and pre-clinal data have challenged this concept [25]. From a pharmacological point of view, palbociclib has similar potency against CDK4 and CDK6, abemaciclib and ribociclib have greater potency against CDK4, and abemaciclib is considered the most potent CDK4/6i overall. Additioanlly, abemaciclib inhibits the CDK9 in vivo, which may explain its efficacy as monotherapy and its particular gastrointestinal toxicity [26-28].

The moderate heterogeneity found in the assessment of PFS can be due to the different CDK4/6i used in each trial. Indeed, the clinical improvement in PFS was more pronounced in those trials using abemaciclib or ribociclib in the experimental arm, in contrast with the PACE and PALMIRA trials that continue the same CDK4/6i (palbociclib) and switch ET after progressive disease. For this reason, some authors do not recommend the continuation of this agent plus ET after progression on prior palbociclib treatment since these two randomized trials did not improve the efficacy outcome of PFS [23]. Previous studies have suggested that, despite comparable results in terms of clinical efficacy, currently approved CDK4/6i present relevant pharmacological differences at least in terms of potency, CDK4:CDK6 inhibition ratio, and lipophilicity that might alter clinical outcomes [29]. A remained clinical question to be solved in future trials should address if switching the ET and changing the CDK4/6i provides a better clinical benefit instead of maintaining the CDK4/6 inhibition with the same pharmacological agent.

Variations in the proportion of patients with endocrine-sensitive disease may also be considered as a potential source of heterogeneity in this meta-analysis. Previous studies have shown that patients with endocrine-resistant disease can experience less benefit from CDK4/6i [7,23]. Nevertheless, after subgroup analysis, we did not find any significant difference in the assessment of PFS according to previous response to ET, challenging the idea of previous response as a predictive marker for subsequent therapies in HR+/HER2- metastatic BC.

Although the comparative arms in the included clinical trials employed fulvestrant as ET in most patients, 10-17% of these individuals were treated with letrozole or exemestane alone. However, we consider that this fact should not introduce a significant source of bias due to the proven similar efficacy of fulvestrant and exemestane in patients with previously treated HR+/HER2- metastatic BC. After a systematic review, we found that fulvestrant or aromatase inhibitors as monotherapy provide minimal benefit in this setting, with a median PFS from 2.8 to 4.8 months.

The rationale for continued CDK4/6i treatment is that patients may progress because of resistance to ET rather than to CDK4/6i [7]. One of such resistance mechanisms is related to acquired mutations in ESR1. Some of the included trials performed subgroup analyses based on this genetic alteration, as well as in individuals with a mutation in the PI3KCA or Akt pathway [7,23]. Pre-specified subgroup analysis performed in the MAINTAIN, PACE, and postMONARCH trials showed that patients harboring mutations in the ESR1 gene or pathogenic variants in the PAM pathway also benefit in terms of PFS of continuing CDK4/6 inhibition. However, the calculation of the pooled HR for PFS in patients with ESR1 wild type or without genetic alteration in PIK3CA, Akt, or PTEN exhibited high heterogeneity (I^2^: 80-85%). Hence, these findings are exploratory and should be validated in a broader population. Moreover, the number of patients detected with these genetic variants was low in the included trials; therefore, these results are hypothesis-generating and warrant more clinical data to establish these or other genetic variants as predictive biomarkers for patients using CDK4/6i.

Another preclinical finding to support the continuation of CDK4/6 inhibition at disease progression relies on the incomplete cross-resistance found between abemaciclib and palbociclib or ribociclib, reinforcing the concept of CDK4/6i rotation, or the use of new CDK inhibitors [23,30]. In line with our findings, retrospective studies and several phase II single-agent clinical trials have consistently shown the benefit of switching ET and continuing CDK4/6 therapy in patients with HR+/HER2- metastatic BC whose disease has progressed on prior hormonal therapy and a CDK4/6i. In fact, the median PFS reported in these cohort studies is very similar to the described in this systematic review [31,32].

The validity of our findings should be regarded cautiously since new therapeutic strategies with targeted therapies have been recently explored in this particular population [11-15]. For example, the phase 3 SONIA trial [33] has challenged the paradigm of HR+/HER2- metastatic BC treatment after showing that first-line treatment with ET and CDK4/6i, followed by fulvestrant, did not improve PFS nor OS in comparison to second-line CDK4/6 inhibition plus fulvestrant after first-line ET. Therefore, for patients using second-line therapy with CDK4/6i and fulvestrant, further studies should clarify the role of continuing CDK4/6 inhibition in disease progression.

Our study has some limitations due to the inclusion of phase II clinical trials with a relatively small sample size and subject to heterogeneities in their design. We also acknowledged that publication bias and the aforementioned potential causes of heterogeneity can contribute to uncertainty in the interpretation of existing data. However, the calculation of the pooled HR through a random effects model assumed this variability and provided a better estimate for the current analysis. Another limitation of our study was the absence of individual data for analysis, and the lack of variables for sensitivity analyses to further explore causes of heterogeneity among trials. Nevertheless, to our knowledge, this is the first systematic review and meta-analysis exploring a relevant clinical question for which available studies have reported conflicting results.

## Conclusions

In conclusion, continuation of CDK4/6 inhibition, with a different agent (as shown in the majority of patients included in the MAINTAIN and postMONARCH trials) and switching ET may be worthwhile in some patients, rather than continuing the same CDK4/6i, as was done in PACE and PALMIRA trials (with palbociclib). Our findings support the hypothesis that changing ET and switching to a different CDK4/6i is a valid strategy to overcome clinical resistance to such therapies.

Future studies should identify molecular biomarkers to better select those patients who can benefit from this approach. This therapeutic strategy can be particularly applied to those patients with no genomic alterations in the PMA pathway (PIK3CA mutation, Akt or PTEN alterations) or in the estrogen signaling (i.e., ESR mutations), for whom new therapies have emerged as valid options. Clinicians must take into account recent phase III clinical trials that have demonstrated a clinical benefit of ET plus targeted therapy combinations for these patients. Similarly, individuals with germline pathogenic variants in BRCA genes can benefit from PARP inhibitors, and patients with HER2 low status can derive some clinical benefit from trastuzumab-deruxtecan after failure on CDK4/6i. Therefore, consideration of the role of CDK4/6i after prior CDK4/6i exposure should be regarded cautiously, taking into account the complex mechanism implicated in therapy resistance to these cell cycle checkpoint inhibitors and considering the expanding available therapies in patients with HR+/HER2- metastatic BC.

## Appendices

Search Strategy

The following terms were used to search for trials, with the appropriate changes to adapt terms to different databases:

1. Breast cancer [tiab]

2. Metastatic breast cancer [MeSH]

3. Hormone-receptor-positive breast cancer cancer [tiab]

4. CDK.4/6 [MeSH]

5. CDK 4/6 inhibitors

6. Progressive disease [MeSH]

7. Abemaciclib [MeSH]

8. Palbociclib [MeSH]

9. Ribociclib [MeSH]

10. #1 OR #2 OR #3

11. #1 AND #4

12. #1 AND #5

13. #2 AND #4

14. #2 AND #5

15. #2 AND #7

16. #2 AND #8

17. #2 AND #9
